# Hemizygosity for Atm and Brca1 influence the balance between cell transformation and apoptosis

**DOI:** 10.1186/1748-717X-5-15

**Published:** 2010-02-22

**Authors:** Fengtao Su, Lubomir B Smilenov, Thomas Ludwig, Libin Zhou, Jiayun Zhu, Guangming Zhou, Eric J Hall

**Affiliations:** 1 Institute of Modern Physics, Chinese Academy of Sciences, Lanzhou 730000, PR China; 2 Center for Radiological Research, Columbia University Medical Center, New York, NY 10032, USA; 3 Institute for Cancer Genetics, Columbia University Medical Center, New York, NY 10032, USA

## Abstract

**Background:**

In recent years data from both mouse models and human tumors suggest that loss of one allele of genes involved in DNA repair pathways may play a central role in genomic instability and carcinogenesis. Additionally several examples in mouse models confirmed that loss of one allele of two functionally related genes may have an additive effect on tumor development. To understand some of the mechanisms involved, we examined the role of monoallelic loss or Atm and Brca1 on cell transformation and apoptosis induced by radiation.

**Methods:**

Cell transformation and apoptosis were measured in mouse embryo fibroblasts (MEF) and thymocytes respectively. Combinations of wild type and hemizygous genotypes for ATM and BRCA1 were tested in various comparisons.

**Results:**

Haploinsufficiency of either ATM or BRCA1 resulted in an increase in the incidence of radiation-induced transformation of MEF and a corresponding decrease in the proportion of thymocytes dying an apoptotic death, compared with cells from wild-type animals. Combined haploinsufficiency for both genes resulted in an even larger effect on apoptosis.

**Conclusions:**

Under stress, the efficiency and capacity for DNA repair mediated by the ATM/BRCA1 cell signalling network depends on the expression levels of both proteins.

## Background

In recent years data from both mouse models and human tumors, suggest that loss of one allele of genes involved in DNA repair pathways may play an important role in carcinogenesis. Haploinsufficiency as a result of loss of allele for APC, ARF, ATM, BRCA1, BRCA2, LKB1, CDKN1B, P53, RB and other proteins has been shown to contribute to tumorigenesis [1-6]. Additionally, several examples in mouse models confirmed that hemizygosity for functionally related genes may have an additive effect on tumor development. Combined hemizygosity for *Xpc *and *p53*, *Atm *and *p53*, and *Fen1 *and *Apc *genes predispose humans to UV radiation-induced skin cancer, mammary carcinoma or adenocarcinomas, respectively [7-9]. Importantly, hemizygous genotypes did not contribute to tumor development alone, but if combined with hemizygosity for another gene involved in DNA repair, the contribution became significant. All of this evidence suggested that tumorigenesis may depend on the expression levels of single or combination of proteins. We have reported that primary mouse cells haploinsufficient for either of two important DNA repair proteins, Atm or Rad9, are more sensitive to transformation by radiation and are less apoptotic when compared with wild-type controls [10]. Furthermore, cells doubly haploinsufficient for Atm and Rad9 showed an even higher level of radiation-induced transformation and an even lower level of apoptosis than those cells haploinsufficient for either one of these proteins alone. We now extend these studies to primary mouse cells derived from animals hemizygous for *Brca1 *and *Atm*. Earlier reports suggested a link between Atm heterozygosity and breast cancer. The reported estimated relative risk varied in the range of 1.5 to 12 fold [11-13]. Different mechanisms by which *ATM *heterozygosity contributes to breast cancer pathobiology were proposed, most of which were associated with the expression of dominant negative ATM protein [14,15]. However a large number of the detected *ATM *mutations in familial breast cancer cases are actually result in truncated gene products resulting in no expression of ATM protein from the mutant allele [13]. The frequency of such mutations is also very high (> 80%) in ATM patients [16,17]. Importantly, the frequency of *ATM *heterozygotes with null mutation for one of the alleles could be as high as 1-3% of the US population [18,19]. Taken together, these observations led us to investigate the effects of monoallelic loss for two genes - ATM and BRCA1 in primary cells for two endpoints: cell transformation and cell apoptosis. Cells matching these criteria were derived from established *Atm *and *Brca1 *heterozygous parental strains of mice. In both parental strains, one of the alleles of the *Atm *or *Brca1 *genes was truncated, resulting in loss of expression of the corresponding protein from the truncated allele. The biological function and roles of ATM and BRCA1 are relatively well established. Both proteins are involved in DNA repair and function as sensor/transducers. ATM is involved in the earliest events in DNA double strand break detection and initiates the activation of several pathways linked to cell cycle checkpoint controls [20]. ATM also recruits DNA repair proteins to sites of DNA damage and, along with BRCA1 is part of supramolecular DNA repair complex comprised of many factors [21]. The phosphorylation of BRCA1 by ATM is an important event in the activation of the S/G2 and G2/M checkpoints [22]. BRCA1 likely plays multiple roles in the mechanisms of physical repair of DNA [23,24]. Mutations of either protein are associated with tumor development. ATM deficiency results in lymphoid malignancies and BRCA1 mutation carriers have 50-85% life risk of developing breast cancer [25]. We hypothesize that the appropriate function of signaling networks that facilitate either DNA damage repair, cell signaling, or programmed cell death, depends on the expression levels of key proteins. Consequently, hemizygosity causing haploinsufficiency may create conditions where network efficiency is reduced leading to decreased effectiveness of DNA repair. In this study we show that hemizygosity for either Atm or Brca1 or both increases the incidence of cell transformation and decreases apoptosis. Remarkably, cells hemizygous for both genes show the lowest levels of radiation-induced apoptosis.

## Methods

### Mice

*Atm *and *Brca1 *heterozygous (+/-) animals have been described previously [26,27]. In both mouse models one of the *Atm *or *Brca1 *alleles have been disrupted by targeted mutagenesis. This mutagenesis prevented any protein synthesis from the targeted alleles. As a result, Atm or BRCA1 proteins were coded only from the wild type alleles. *Atm *and *Brca1 *hemizygous mice were mated and only F1 littermates were used. Genotypes were determined by PCR. The p53 status was "wild type" for both genotypes as shown earlier [27,28]

### Embryo Cell Preparation

Pregnant mice were sacrificed on day 14 of the gestation. Mouse embryo fibroblasts (MEF) from each embryo were cultured separately with DMEM high glucose (Invitrogen) supplemented with 15% FBS (ATCC) and then genotyped. Four genotypes of MEF cells from the same litter were used for each experiment: wild-type, (*Atm*wt/*Brca1*wt), single hemizygous (hz) for *Atm *(*Atm*hz/*Brca1*wt) single hemizygous for *Brca1 *(*Atm*wt/*Brca1*hz) and double hemizygous (*Atm*hz/*Brca1*hz).

### Cell Transformation Assay

Exponentially growing MEFs received a dose of 2 Gy of γ-rays in an acute exposure, and controls were sham-irradiated. MEFs were then plated in 10 cm plates at a density of 6,000 cells/plate over a feeder layer of 70,000 cells prepared from the same embryo but irradiated previously with a supralethal dose of 30 Gy. After 2 weeks of growth in DMEM medium supplemented with 10% fetal bovine serum at 37°C in a 5% CO_2 _air-humidified incubator, cells were fixed, stained, and yields of transformed clones scored. The scoring criteria was developed and examined by preliminary experiments, where embryo cells were irradiated and plated at the same density. The clones which seemed dense and had stellate-shaped piled cells were photographed and isolated with cloning cylinders. These clones were expanded and injected into nude mice. Those that caused the development of subcutaneous tumors were designated as transformed. Clones that matched their shape and dimensions were scored as transformed in later experiments. Plating efficiency, cell surviving fractions, and the spontaneous and radiation-induced frequency of transformation were determined.

### Evaluation of micronuclei

Exponentially growing MEF cells were plated at density of 50,000 cells/well of 12-well plate. Next day, the cells were exposed to various doses of γ-rays. Immediately after irradiation, 1.5 μg/ml of cytochalasin B (Sigma) was added to each well. 24 hours later, the cells were fixed with acetic acid and methanol (v/v = 3:1), and stained with 3 μg/ml of acridine orange (Sigma) for 1 min. Micronuclei in binucleated cells (BN) were counted under fluorescent microscope. More than 500 BN cells were scored for each sample.

### Apoptosis assay

Mice were irradiated with 5 Gy of γ-rays. 24 hours later, thymuses from the irradiated and sham-irradiated control mice were isolated, weighed and homogenized gently for single cell suspension preparation. After estimation of the total cell number, 1× 10^6 ^cells from each genotype were labeled with CD^4+ ^and CD^8+ ^specific antibodies (Pharmingen) and two color flow cytometry analysis was used to estimate the survival of each thymocyte subtype. Total of 20,000 cells for each genotype were examined and the percent of double positive CD^4+^/CD^8+ ^cells was estimated based on that number.

### Comet Assay

DNA damage and repair were evaluated with alkaline comet assay according to the report by Olive et al [29] with some modifications. Single MEF cells were harvested by trypsin treatment and resuspended in DMEM containing 10% FBS at a concentration of 1×10^6 ^cells/ml. An aliquot of 100 μl cell suspension was mixed with 300 μl 0.5% low melting-point agarose (Amresco) in DMEM containing 10% FBS. 100 μl of the mixture was layered on glass slide pre-coated with 0.5% LE agarose and covered with another glass slide. After brief incubation on ice for agarose solidification, the cover slides were carefully removed and the samples were gently immersed into freshly prepared lysis solution (2.5 M NaCl, 10 mM Tris, 1% sodium lauryl sarcosinate, 100 mM EDTA, 1% Triton-100, and 10% DMSO) for 1.5 hrs followed by incubation for 20 min in electrophoresis buffer (1 mM EDTA, 300 mM NaOH, pH > 13). The electrophoresis was performed in the same buffer (20 min, 20 V, 300 mA). The samples were neutralized with 0.4 M Tris-HCl buffer (pH 7.5) and air-dried after a brief fixation with 70% ethanol.

Individual cells were visualized with BrdU staining and photographed under fluorescence microscope. 100 comets of each sample were analyzed with a free software called Casp [30].

## Results

### Cell Transformation Assay

Radiation-induced transformation of MEF was examined as a surrogate for carcinogenesis in vivo. A total of 19 embryos from five litters were used and included the following genotypes: *Atm*wt/*Brca1*wt, *Atm*wt/*Brca1*hz, *Atm*hz/*Brca*1 wt and *Atm*hz/*Brca1*hz. Yields of transformed clones were measured both for unexposed controls and after a dose of 2 Gy. The results shown in Tables 1 and 2 indicate a statistically significant increase in transformation frequency for the single and doubly hemizygous cells. Transformation frequencies for these cells were nearly two times higher than the one of wild-type cells. *Brca1 *hemizygotes show a similar transformation frequency as the *Atm *hz, however, the interesting point to note is that the double hemizygotes *Atm/Brca1*, show little or no increase over *Brca1*hz or *Atm *hz alone. There were small statistically not significant differences in the clonogenic survival for all populations after irradiation (results not shown).

**Table 1:** Transformation frequencies of unirradiated or irradiated cells differing in the status of *Atm *and *Brca1*.

Genotype	Dose (Gy)	Total number of clones scored	Number of transformed clones	Transformed clones (%)
*Atm*wt/*Brca1*wt	0 Gy	31220	7	0.02
	
	2 Gy	21880	26	0.12

*Atm*wt/*Brca1*hz	0 Gy	34380	11	0.03
	
	2 Gy	17142	32	0.19

*Atm*hz/*Brca1*wt	0 Gy	34170	11	0.03
	
	2 Gy	16720	36	0.21

*Atm*hz/*Brca1*hz	0 Gy	26660	9	0.04
	
	2 Gy	12046	27	0.22

**Table 2:** Comparisons of radiation induced transformation between MEFs of different genotypes vs. wild type MEFs.

	*Atm*hz/*Brca1*wt	*Atm*wt/*Brca1*hz	*Atm*hz/*Brca1*hz
Relative transformation (2 Gy)	1.8	1.66	1.88

t-test	P = 0.03	P = 0.05	P = 0.018

Relative transformation is defined as the ratio of the number of transformed clones per surviving hemizygous cells relative to the number of transformed clones per surviving wild type cells. The statistical significance of differences in transformation frequency between the various cells with hemizygous genotypes and wild type cells was analyzed by the Student's t-test.

### Background DNA damage estimation in the different genotypes

**Figure 1:**
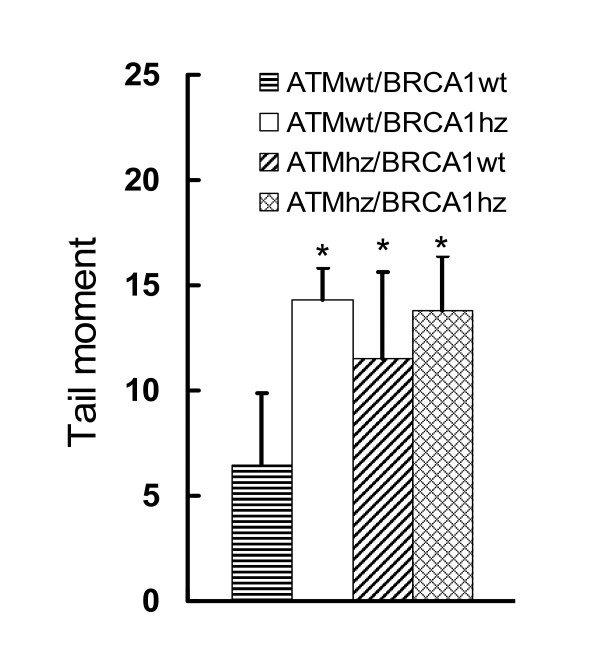
**DNA damage measured with alkaline comet assay**. Total DNA damage measured with alkaline comet assay points to the higher background DNA damage in the hemizygous genotypes. The data is from three independent experiments where total of 100 cells/genotype were scored.

In these experiments we accessed the background DNA damage in all four genotypes by alkaline comet assay (Figure 1). Notably there were statistically significant differences in the tail moments between the wild type and all hemizygous genotypes. These differences illustrate that cells that are singly or doubly hemizygous for *Atm *and *Brca1 *have more background DNA damage than wild type cells. This elevated background of DNA damage may point to the higher vulnerability of these cells to DNA damage and cell transformation if additional damage is induced.

### Micronucleus Assay

**Figure 2:**
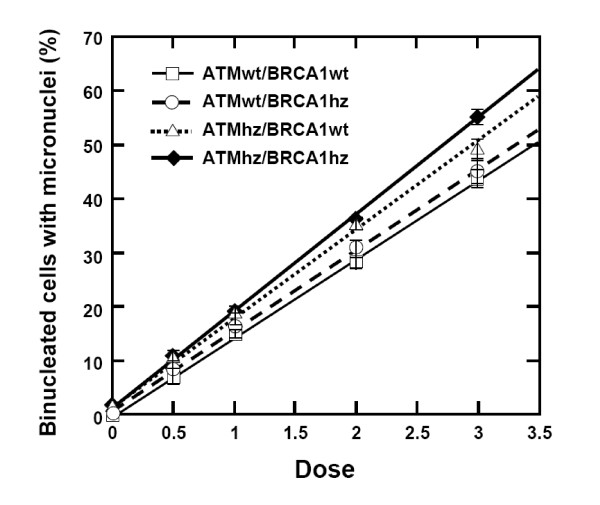
**Induction of micronuclei by graded doses of radiation**. Induction of micronuclei by graded doses of radiation in mouse embryo fibroblasts having different genetic backgrounds. Data are shown as a mean and standard error from 3 independent experiments. At a dose of 3 Gy of γ-rays, there is a statistically significant difference between the double hemizygous and the other genotypes.

Figure 2 shows the data for micronuclei, scored in binucleated cells, 24 hours after exposure to graded doses of 0.5 to 3 Gy of γ-rays. There was a statistically significant increase of micronuclei in cells hemizygous for both *Atm *and *Brca1 *at the highest dose, but for lower doses no such differences were found. These results suggests that the DNA damage induced by radiation is less efficiently repaired in double hemizygous cells and may point to an increased mutation accumulation in these cells after DNA damage is induced.

### Apoptosis of Thymocytes

**Figure 3:**
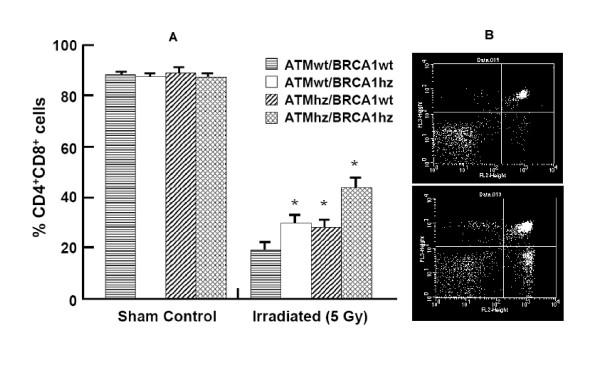
**CD4^+ ^CD8^+ ^cell survival after γ-ray irradiation**. A) CD4^+ ^CD8^+ ^cell survival after γ-ray irradiation. Cell survival was highest (apoptosis was lowest) in the double hemizygous background. In contrast the percent of CD4^+ ^CD8^+ ^cells does not depend of the genotype in nonirradiated cells. The numbers of mice used was three per genotype for the controls and five per genotype for the irradiated mice. B) Representative image of flow cytometry of the thymocytes. Top panel: *Atmwt/Brca1wt *genotype. CD4^+ ^CD8^+ ^cells appear at the upper right quadrant and are 31% of the total cell numbers. Note also the very low numbers of CD4^+ ^and CD8^+ ^cells which appear in the lower right and upper left quadrants. Lower panel represents *Atmhz/Brca1hz *genotype where 61% of the double positive CD4^+ ^CD8^+ ^cells survived accompanied also with high numbers of CD4^+ ^and CD8^+ ^cells (lower left and upper right quadrants).

We examined the survival of the most numerous type of cells in the thymus (more than 80% of all cells), CD4^+^/CD8^+ ^thymocytes, after in vivo γ-irradiation (Figure 3). As expected, 24 hrs after irradiation the numbers of CD4^+^/CD8^+ ^cells were significantly reduced. The survival of CD4^+^/CD8^+ ^cells from single *Atm *hemizygous mice was 10% higher than the wild type controls. Interestingly, the survival of *Brca1 *hemizygous thymocytes trends similarly. However, compared with the other three genotypes, the survival of the double hemizygous thymocytes was significantly higher. More than 40% of these thymocytes survived which shows that they are more resistant to radiation and less apoptotic than the other genotypes examined. This implies that *Atm/Brca1 *cells may accumulate mutations at a higher rate than the other genotypes.

## Discussion

This study demonstrates that cells hemizygous for either Atm or Brca1 are more sensitive to transformation by radiation and exhibit defective induction of apoptosis under stress. Remarkably, combined hemizygosity for both genes show additive negative effect on apoptosis induction and increased genomic instability reflected by micronuclei formation.

In recent years, epidemiological data as well as studies in mouse models confirmed that heterozygosity may play a significant role in tumor initiation and development. The most striking conclusion from these experiments is that heterozygosity for a single gene may contribute to tumor formation. To what degree this may reflect in increased cancer risk heterozygous carriers is a very important issue which can be resolved only after understanding the mechanisms underlying the role of heterozygosity in tumor formation. The role of heterozygosity is more obvious in cases where the product of the mutant allele is a truncated protein having dominant negative effect. Truncated versions of P53, Rb, Ras, NF1, ATM, BRCA1 and 2, INK4 family of proteins, CREB binding protein (CBP) and others have been identified in different tumors [31,32]. Much more difficult to explain are the instances where the mutant allele does not produce any protein. Cumulative data acquired in cases where the role of heterozygosity of a gene (one allele inactivated, no protein expression from it) was studied in mouse models, show that more than twenty genes could be implicated in tumor development [33]. A subset of these 20 genes is included in the group of the 300 known cancer genes [34]. Many of these genes maintained their hemizygous status in the tumors that developed as a result of their hemizygosity. In general, the only difference between the wild type and hemizygous status of these genes was the haploinsufficiency for the corresponding protein.

We hypothesize that haploinsufficiency is a factor mostly in acute cell conditions, where different factors trigger stress response pathways. Due to the networked nature of this response, the insufficient expression level(s) of some proteins may lead to reduced overall network response. As a consequence, stress related processes, apoptosis for example, may be less effective. Previously, we substantiated this idea using a system where both *Atm *and *Rad9 *genes were haploinsufficient [10]. In the current study we used another pair of DNA repair genes - *Atm *and *Brca1*. As was the case in our prior study, the background transformation frequency of MEF was the same for all studied genotypes. Remarkably, the transformation frequency after induced DNA damage was dependant on the genetic background. Both hemizygous genotypes show statistically significant increases in cell transformation in comparison with the wild type cells. Interestingly, the transformation frequency of MEF on a doubly hemizygous background was in the same range as the singly hemizygous MEF which indicated that there is no additive effect of hemizygosity for *Atm *and *Brca1 *genes for this endpoint. Nevertheless, these results confirm that stress related pathways may depend on proper expression levels of these key proteins.

The induction of genomic instability was monitored by measuring micronuclei (MN) formation. In one set of experiments, we determined the induction of MN in different genotypes. Our results show that combined hemizygosity for *Atm *and *Brca1 *genes results in elevated levels of MN. This observation supports the conclusion from the transformation experiments and indicated strongly that processes active under stress depend on the expression levels of both Atm and Brca1 proteins.

The induction of cell transformation is thought to depend on the efficiency of apoptosis induction. In order to estimate the role of genetic background in apoptosis induction, and since ATM plays very important role in thymocyte apoptosis after irradiation [35], we measured the survival of thymocytes *in vivo *after radiation induced DNA damage. Under the conditions we used, cell survival depended largely on the genetic background. We registered the highest level of cell survival in the doubly hemizygous cells, where the rates were two fold greater in wild type cells and 1.5 fold greater than singly hemizygous cells. Since statistically, the number of damaged sites per cells should be the same for all genotypes, the differences in cell survival suggests that damage detection was less efficient in the double heterozygous cells and that more cells with DNA damage may accumulate in the thymuses of double heterozygous animals. Many if these cells will undergo apoptosis in subsequent division attempts but a very small fraction may survive increasing the probability of subsequent transformation.

The results from the estimation of the background DNA damage done by alkaline comet assay were somewhat unexpected for us. They clearly show that the background DNA damage is higher in the hemizygous genotypes. Since we didn't find difference in the background transformation frequency (and apoptosis, although apoptosis was measured in different cell type) between the heterozygous and wild type genotypes, we may conclude that the DNA damage detected by this method is not relevant to the background transformation frequency. It could be related though to the highest transformation levels in the heterozygous genotypes after irradiation where the combination of this damage and the one induced by radiation may result in higher degree genetic instability.

Considering the network of physical interactions between active factors in living cells may help to explain how it is that reduced levels of expression of a single protein may have such a large effect in the system of events which comprise the biology of the cell. Biological networks are capable of self assembly and disassembly. For example, many local networks may be assembled only when they are needed - for instance after DNA double-strand breaks are induced. The requirement for assembly in response to an event at an unknown point in a relatively large (on molecular scale) area, introduces spatial and quantitative limitations on the process. DNA double-strand breaks are a local event that may appear at any place in the nucleus. A local network has to be assembled at the points of DNA double-strand breaks in order to signal and initiate the repair. Proteins, potential members of the local networks, have to be in close proximity to the break or to be able to translocate quickly to the site. Several experiments confirmed that this is the case. Immunofluorescence analysis of cells after radiation induced DNA double-strand breaks show that many DNA repair proteins, like ATM, P53BP1, MRE11, Rad50 and NBS1, ATR, colocalize and form discrete foci on the sites of DNA damage [36,37]. In addition, migration of DNA repair proteins toward the site of DNA damage has been analyzed by FRAP. By measuring the diffusion coefficient of various repair proteins it has been shown that translocation and transient immobilization of RAD51, RAD52, RAD54 as well as the NER repair complex ERCC1-XPF and P53BP1 [38-40] occurs at DNA repair sites in mammalian cells. In the case of multiple DNA dsb, haploinsufficiency for ATM or BRCA1 may lead to incomplete assembly of the repair complex. As a result, some DNA dsb may not be detected or repaired and the cells will not fail to correctly undergo apoptosis. In this way, the failure of local networks could lead to the accrual of mutations in living cells.

## Conclusions

In summary, we have shown that hemizygosity and combined hemizygosity for Atm and BRCA1 both constitute a prominent contribution to radiation induced cell transformation and apoptosis. While it has long been hypothesized that radiosensitivity in some individuals may well be the result of haploinsufficiency for low penetrance genes, little progress has been made in elucidating specific examples. We have now identified three genes with high penetrance and a low frequency of mutation that confer sensitivity to radiation induced effects, such as cancer. This is relevant given that the frequency of mutation of any individual sensitizing gene inducing heterozygosity among individuals in the general human population may be low and largely undetected. Compound heritable mutations inducing heterozygosity in more than one radio sensitizing gene could render a sub-population particularly radiosensitive. Since such heritable mutations can become concentrated in certain ethnic groups, elements of the human population may be especially vulnerable to radiation induced biological effects.

## Competing interests

The authors declare that they have no competing interests.

## Authors' contributions

LBS and TL provided the mice, mating, genotyping, embryo cells isolation and culture. FS and LZ and GZ carried out the comet assay, transformation assays, apoptosis and micronuclei assay. EJH conceived the study and participated in its design and coordination. LBS and EJH drafted the manuscript. All authors read and approved the final manuscript.
